# Transthyretin familial amyloid polyneuropathy (TTR‐FAP): Parameters for early diagnosis

**DOI:** 10.1002/brb3.889

**Published:** 2017-12-19

**Authors:** Fabiola Escolano‐Lozano, Ana Paula Barreiros, Frank Birklein, Christian Geber

**Affiliations:** ^1^ Department of Neurology University Medical Center of the Johannes Gutenberg University Mainz Mainz Germany; ^2^ German Association for Liver Transplantation Mainz Germany; ^3^ Red Cross Pain Center Mainz Germany

**Keywords:** amyloidosis, autonomic function, neurophysiology, polyneuropathy, TTR‐FAP

## Abstract

**Background:**

Familial transthyretin amyloidosis is a life‐threatening disease presenting with sensorimotor and autonomic polyneuropathy. Delayed diagnosis has a detrimental effect on treatment and prognosis. To facilitate diagnosis, we analyzed data patterns of patients with transthyretin familial amyloid polyneuropathy (TTR‐FAP) and compared them to polyneuropathies of different etiology for clinical and electrophysiological discriminators.

**Methods:**

Twenty‐four patients with TTR‐FAP and 48 patients with diabetic polyneuropathy (dPNP) were investigated (neurological impairment score NIS; neurological disability score NDS) in a cross‐sectional design. Both groups were matched for gender and presence of pain. Quantitative sensory testing (QST), sympathetic skin response (SSR), heart rate variability (HRV), and nerve conduction studies (NCV) were performed. Both groups were compared using univariate analysis. In a stepwise discriminant analysis, discriminators between both neuropathies were identified. These discriminators were validated comparing TTR‐FAP patients with a cohort of patients with chemotherapy‐induced polyneuropathy (CIN) and chronic inflammatory demyelinating neuropathy (CIDP).

**Results:**

TTR‐FAP patients scored higher in NDS and NIS and had impaired cold detection (CDT,* p* = .024), cold–warm discrimination (TSL,* p* = .019) and mechanical hyperalgesia (MPT,* p* = .029) at the hands, SSR (upper limb, *p* = .022) HRV and ulnar and sural NCS (all *p* < .05) were more affected in TTR‐FAP. Ulnar nerve sensory NCV, CDT, and the MPT but not the other parameters discriminated TTR‐FAP from dPNP (82% of cases), from CIN (86.7%) and from CIDP (68%; only ulnar sNCV).

**Conclusion:**

Low ulnar SNCV, impaired cold perception, and mechanical hyperalgesia at the hands seem to characterize TTR‐FAP and might help to differentiate from other polyneuropathies.

## 1 INTRODUCTION

Transthyretin amyloidosis is caused by a mutation in the transthyretin gene. As a result, amyloid fibrils accumulate and cause function impairment of different organs and tissues, particularly in the peripheral nerves leading to polyneuropathy (TTR‐FAP). Frequently, neuropathy is the first symptom, often starting in young adulthood. If untreated, the progressive organ dysfunction causes death within about 10 years. However, early treatment increases life span (Hund, Linke, Willig, & Grau, 2001).

The amyloid accumulation in the peripheral nerves causes distal symmetrical polyneuropathy with neuropathic pain and sensory loss, autonomic dysfunction (e.g., fainting, gastrointestinal symptoms), and motor impairment (Andrade, 1952). It is controversial whether there is preferential damage to small sensory or autonomic fibers (Said, Ropert, & Faux, 1984), or less selectively to all nerve fibers (Heldestad & Nordh, 2007).

Although breakthroughs in gene therapy are anticipated in the near future (Ueda & Ando, 2014), current causal treatments include liver transplantation and amyloid‐stabilizing agents, which are most effective when initiated at early disease stages (Ando et al., 2013). Therefore, early diagnosis is crucial for a better outcome. However, because of the rarity of the disease and the initially unspecific symptoms, misdiagnoses are frequent and the recognition of TTR‐FAP may be delayed by years (Adams et al., 2016).

Therefore, there is an unmet need for a simple clinical test, or a combination thereof, which alerts physicians to the possibility of TTR‐FAP and makes them take further diagnostic steps such as genetic testing or a biopsy. We looked for a “signature” of TTR‐FAP and applied a battery of clinical and electrophysiological tests to characterize TTR‐FAP and compared these results to patients with common types of polyneuropathy.

## 2 PATIENTS AND METHODS

### 2.1 Standard protocol approvals, registrations, and patient consents

The study was approved by the local ethics committee, and written informed consent was obtained from all patients participating in the study.

### 2.2 TTR‐FAP and diabetic polyneuropathy patients (dPNP)

In a cross‐sectional design, we first analyzed 72 patients from our outpatient clinic: Twenty‐four patients suffered from transthyretin amyloidosis with the following mutations: Val30Met (*n* = 17), Glu89Lys (*n* = 2), Thr49Ala, TTR‐WT, Leu58His, Asp18Glu, and Val20Ile (each *n* = 1). Patients with TTR‐FAP were assessed either at the initial visit after diagnosis (*n* = 11) or when the first symptoms occurred before the disease had been confirmed (*n* = 13) (see Table 1). Forty‐eight patients with type II diabetes were selected from a dPNP cohort between 2009 and 2012 and matched 2:1 by gender and presence of pain to the TTR‐FAP cohort. For all patient groups, selection criteria were based on meeting the diagnostic criteria for dPNP (England et al., 2005) or having a positive genetic test and signs or symptoms of a TTR‐FAP, and age >18 years. In the TTR‐FAP cohort, no patients with diabetes or receiving chemotherapy were included. dPNP patients receiving chemotherapy were also not included.

**Table 1:** Patient characteristics and descriptive statistics of the selected polyneuropathy cohorts (TTR‐FAP, dPNP, CIN, and CIDP)

	TTR‐FAP	dPNP	CIN	CIDP
Total of patients	24	48	48	8
Male gender (number, %)	14 (58%)	30 (58%)	15 (31.3%)	8 (100%)
Below 50 y old, cases (%)	18 (75%)	5 (10.4%)	13 (27.1%)	6 (75%)
Pain, cases (%)	13 (54.1%)	26 (54.1%)	28 (58.34%)	0
Age at first evaluation (y)	46.0 ± 2.8	66.7 ± 1.6	54.1 ± 1.5	47.1 ± 4.2
Median (range)	42.5 (31–74)	67.5 (46–86)	55.5 (26–77)	48.5 (25–61)
Duration from symptom onset to assessment (mo)	14.7 ± 5.4	69.3 ± 8.2	21.1 ± 2.0	12.0 ± 5.3
Median (range)	6 (0–72)	63 (8–182)	16 (4–76)	6.5 (3–48)
NIS‐UL	3.7 ± 0.7	2.1 ± 0.4	n.d	n.d
NIS‐LL (Total)	17.2 ± 4.2	6.7 ± 0.5	10.1 ± 0.54	18.3 ± 1.9
Muscle weakness	8.6 ± 2.5	2.0 ± 0.3	0.5 ± 0.3	6.8 ± 1.8
Reflexes	2.4 ± 0.6	0 ± 0	3.4 ± 0.3	8 ± 0
Sensory symptoms	5.5 ± 1.1	4.5 ± 0.4	6.2 ± 0.2	3.5 ± 0.8
NDS	4.4 ± 0.7	6.6 ± 0.3	7.2 ± 0.3	5.3 ± 0.7
NDS (<3): no deficits (%)	24	0	0	0
NDS (3–5): light deficits (%)	33.3	23.8	10.4	37.5
NDS (6–8): middle deficits (%)	33.3	68	75.0	62.5
NDS (9–10): high deficits (%)	9.5	11	14.6	0

NDS, neurological disability score; NIS, neurological impairment score; CIN, chemotherapy‐induced polyneuropathy; CIDP, chronic inflammatory demyelinating neuropathy.

### 2.3 Validation cohorts

Patients with chemotherapy‐induced polyneuropathy (CIN) and chronic inflammatory demyelinating polyneuropathy (CIDP) were used for validation of the results of the discriminant analysis (see below):

Pain‐matched data sets from 48 patients with CIN were investigated. This group is a subgroup of the CIN patient cohort described before (Geber et al., 2013). CIN patients with diabetes were not included. As TTR‐FAP can clinically mimic CIDP (Rajabally, Adams, Latour, & Attarian, 2016), we investigated eight (unmatched) CIDP patients as a second validation cohort (see Table 1). Those patients matched the diagnostic CIDP criteria of the European Federation of Neurological Societies (Hughes et al., 2006) and did not have diabetes or receive chemotherapy.

### 2.4 Clinical examination

Patients were examined by experienced physicians (FEL, FB, and CG) and validated by a second examiner. The neurological impairment score for cranial nerves, thorax, and both upper and lower limbs (NIS: 0–244 points) (Dyck et al., 1995) and the neurological disability score (NDS: 1–10 points) (Dyck, 1988) were calculated. The NDS was calculated from examination of vibration perception, and pinprick and temperature perceptions in the great toe, and the presence or absence of ankle reflexes. NIS subscores for the upper (NIS‐UL: 0–100 points) and lower limbs (NIS‐LL: 0–88 points) were assessed, and the motor items of the NIS were separately summed up to grade whole body muscle status (0–192 points). The combination of both scores allows a complete neurological evaluation including motor and sensory nerve function (mediated by large and small fiber function). TTR‐FAP patients were further classified according to the polyneuropathy disability score (PND) (Yamamoto et al., 2007).

### 2.5 Technical investigations

Neurophysiological tests were chosen in order to evaluate the function of both small somatic and autonomic fibers (QST, autonomic testing) as well as large nerve fibers (QST, nerve conduction studies).

#### 2.5.1 Quantitative sensory testing (QST)

Quantitative sensory testing was performed on the right foot and hand dorsum according to protocol of the German Research Network on Neuropathic Pain (see [Rolke et al., 2006] for details). In brief, QST assesses sensory loss (hypesthesia, hypalgesia) and gain (hyperalgesia, allodynia) for thermal and mechanical stimuli in comparison with age‐ and sex‐matched normal values. For further details, see Table S1.

#### 2.5.2 Autonomic function tests (AFT)

The sympathetic skin response (SSR) and the heart rate variability (HRV) were evaluated using the FAN^®^‐device (Schwarzer, Munich, Germany) as described previously (Haegele‐Link, Claus, Ducker, Vogt, & Birklein, 2008). Results are compared to normal values from our own laboratory (Haegele‐Link et al., 2008).

##### SSR

In our study, the SSR reflects the activation of sweat glands in response to a loud noise (Shahani, Halperin, Boulu, & Cohen, 1984). The SSR was recorded with electrodes on the hand (and foot) dorsum and on the corresponding sites of palm and sole. Peak‐to‐peak amplitude was assessed. The SSR amplitude is an approximation of sudomotor nerve fiber integrity. The SSR latency was not analyzed because sudomotor fibers are very slowly conducting, and there is no pathological correlate like fiber loss for a further slowing (Toyokura, 2009). Due to the large physiological variability of the SSR, the definition of a normal range of the SSR amplitude is disputable. Therefore, the SSR is regarded “normal” if it can be reliably elicited (amplitude >0 μV) (Spitzer, Lang, Birklein, Claus, & Neundorfer, 1997).

##### Heart rate variability

Cardiac autonomic neuropathy was assessed using heart rate variability (HRV). Seven statistical values in the time domain were obtained at rest, during deep respiration, and the Valsalva maneuver (Kramer, Rolke, Hecht, Bickel, & Birklein, 2005).

#### 2.5.3 Nerve conduction

Motor (tibial, peroneal, and ulnar) and sensory (sural and ulnar) nerve conduction (NCV) was assessed on the right body side. Measurements in the median nerve were not included, due to high prevalence of compression syndromes affecting this nerve both in polyneuropathy patients (especially diabetic and TTR‐FAP) (Ando et al., 2013; Phalen, 1966) as well as in the otherwise healthy population (Atroshi et al., 1999). Measurements were performed according to our laboratory standards (Hopf & Röder, 1996). Amplitudes of muscle compound action potentials (MCAP) were measured peak to peak and sensory nerve action potentials (SNAP) baseline to peak. Reference data were taken from our laboratory (Hopf & Röder, 1996).

### 2.6 Statistical analysis

Data were transferred into *Z*‐scores to compare parameters regardless of their physical units (Gauss, [Link]). Absolute reference data adjusted for age and gender (19, 46) were used to normalize test results of individual patients: *Z*‐score = (single value_test area_ − mean_reference_)/standard deviation_reference._

*Z*‐scores >0 indicate an “increased function” (relevant for QST) compared to controls, whereas *Z*‐scores <0 indicate loss of function. In a sensitivity analysis (without correction for multiple comparisons), Mann–Whitney *U* tests were performed. *Z*‐transformed group data (TTR‐FAP, dPNP) were compared to the reference data using two‐sided independent *t* tests (Magerl et al., 2010). Statistical significance was defined as *p* < .05. Missing values were not considered. All data are presented as mean ± *SEM*.

Stepwise discriminant analyses (method: minimize Wilk's lambda) were calculated to identify predictive discriminators between TTR‐FAP and dPNP. Classification was achieved by calculating Fisher's canonical linear discriminant function (Mclachlan, 1980). The CIN and CIDP cohorts were used as independent samples for confirmation of the discriminant function.

## 3 RESULTS

### 3.1 Patients

TTR‐FAP patients (*n* = 24) were recruited between 2005 and 2012. Patients presented with PND‐score stage I (25%, *n* = 6) and II (75%, *n* = 18); all of them could walk without assistance; 54.1% (*n* = 13) reported neuropathic pain. In 51.4% (*n* = 13) of the cases, a positive family history was known. Patients with diabetes were older (*p* < .001). Motor impairment was uncommon in patients with diabetes (10/24 TTR‐FAP vs. 1/48 dPNP). The duration of the disease by the time of assessment was longer in the diabetic group (TTR‐FAP: 14.7 ± 5.4 months; dPNP: 69.3 ± 8.2 months; *p* < .001).

In the CIN cohort (*n* = 48), 58.8% of the patients reported pain; in the CIDP cohort (*n* = 8) all patients were men. None of them reported pain (see Table 1 for further details).

#### 3.1.1 Clinical scores

The total muscle weakness score was generally low. It was higher in TTR‐FAP compared to dPNP patients (TTR‐FAP: 13.6 ± 4 vs. dPNP: 0.1 ± 0.1; *p* < .001). In contrast, the NDS was higher in the dPNP group (TTR‐FAP: 4.4 ± 0.3 vs. dPNP: 6.6 ± 0.3 *p* = .008). The more impaired function of the limbs in TTR‐FAP is reflected by the higher NIS‐UL (TTR‐FAP: 3.7 ± 0.7 vs. dPNP: 2.1 ± 0.4, *p* = .035) and NIS‐LL (TTR‐FAP: 16.5 ± 4.1 vs. dPNP: 6.7 ± 0.5). Higher NIS‐scores were due to higher muscle weakness (*p* = .01) and weaker reflexes (*p* = .001), while sensibility scores were similar in both groups (Table 2).

**Table 2:** Results of the clinical examination. While NDS was increased in dPNP, TTR‐FAP patients showed more muscle weakness and a higher NIS‐LL score (*p < .05, **p < .01, ***p < .001)

		*n*	Mean	*SEM*	
NDS	dPNP	47	6.6	0.3	**
	TTR‐FAP	21	4.4	0.7	
Muscle weakness	dPNP	48	0.1	0.1	***
	TTR‐FAP	24	13.6	4.1	
NIS‐LL	dPNP	48	6.7	0.5	*
	TTR‐FAP	24	16.5	4.1	
NIS‐UL	dPNP	48	2.1	0.4	*
	TTR‐FAP	24	3.7	0.7	
NIS (Total)	dPNP	48	8.8	0.9	**
	TTR‐FAP	24	20.9	4.8	

NDS, neurological disability score; NIS, neurological impairment score.

#### 3.1.2 QST

**Figure 1:**
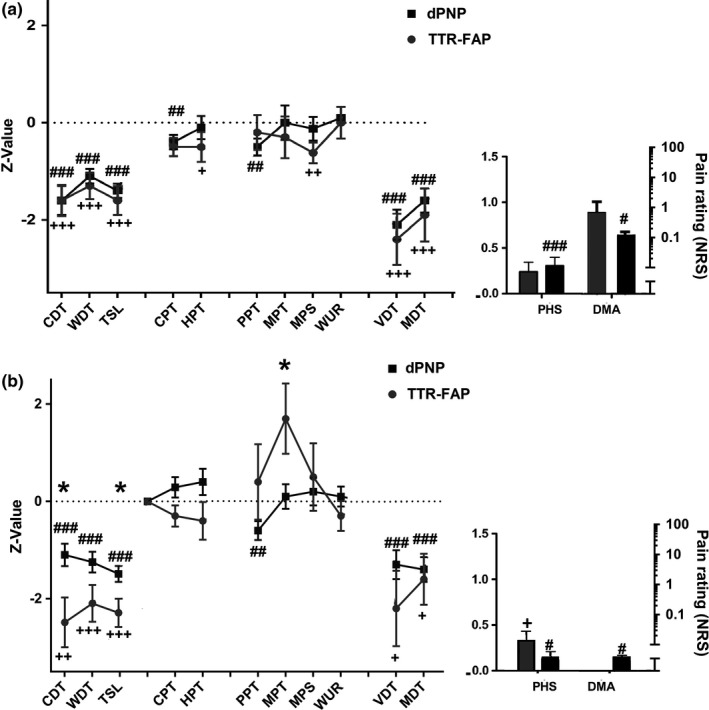
Results of the quantitative sensory testing (QST) in the foot (a) and in the hand (b). Significant differences between TTR‐FAP and dPNP are indicated by * (*p < .05; **p < .01; ***p < .001); significant differences between dPNP and a normal population are indicated by # (^#^ *p* < .05; ^##^ *p* < .01; ^###^ *p* < .001); and significant differences between TTR‐FAP and a normal population are indicated by + (^+^ *p* < .05; ^++^ *p* < .01; ^+++^ *p* < .001). (a) In the foot, we observed differences in several parameters when comparing dPNP patients to a normal population. CDT, WDT, and TSL as well as CPT, VDT, and MDT were pathological in both groups, whereas PPT, PHS, and DMA were significantly pathological only in dPNP and MPS only in the TTR‐FAP cohort. The phenotypical comparison of both groups by QST showed no significant differences when testing on the foot. (b) When the hand was chosen as test area, pathological values were registered for CDT, WDT, TSL, and PHS in both groups compared to a normal cohort. Moreover, the dPNP group showed impairment of PPT and DMA. When comparing both groups, significant differences in CDT and MPT between dPNP and amyloidosis were shown (*), pointing to a more severe impairment in the cold detection and mechanical pain threshold in the group of patients with TTR‐FAP

On the foot dorsum, in both TTR‐FAP and dPNP patients, the majority of QST parameters had lower *Z*‐scores compared to normal values. This indicates loss of function as the common QST pattern in both neuropathies. There were no differences between TTR‐FAP and dPNP (Figure 1a). On the back of the hand, cold detection thresholds (*z* = −2.5 ± 0.5, vs. *z* = −1.1 ± 0.2, *p* = .024) and thermal sensory limen (*z* = −2.3 ± 0.3 vs. −1.5 ± 0.2, *p* = .019) were more impaired in TTR‐FAP. TTR‐FAP but not dPNP patients were presented with mechanical hyperalgesia (MPT, *z* = 1.7 ± 0.7 vs. *z* = 0.1 ± 0.3, *p* = .029) (Figure 1b).

#### 3.1.3 Nerve conduction

Sural sensory NCV (SNCV) could be obtained from 13 TTR‐FAP and 25 dPNP; SNCV was lower in dPNP (TTR‐FAP: *z* = −1.6 ± 0.9 vs. dPNP: *z* = −3.3 ± 0.4, *p* = .015). Ulnar SNCV was measurable in 20 TTR‐FAP and 41 dPNP patients. The SNCV was markedly reduced in the TTR‐FAP group (TTR‐FAP: *z* = −5.5 ± 1.1 vs. dPNP: *z* = −1.0 ± 0.2, *p* < .001).

The remaining NCS studies were not different between groups (Table 3).

**Table 3:** Results of the NCS. The SNCV of the ulnar nerve was found to be significantly reduced in TTR‐FAP, while the nerve conduction studies nerve conduction studies (NCV) of the sural nerve was found to be more impaired in the dPNP group (*p < .05, ***p < .001)

			*n*	Mean	*SEM*	
Ulnar N. Sens.	Amplitude	dPNP	42	−1.5	0.1	n.s.
		TTR‐FAP	20	−1.2	0.3	
	NCV	dPNP	41	−1.0	0.2	***
		TTR‐FAP	20	−5.5	1.1	
Ulnar N. Mot.	Latency	dPNP	44	−0.9	0.2	n.s.
		TTR‐FAP	21	−1.7	0.7	
	Amplitude	dPNP	44	0.4	0.1	n.s.
		TTR‐FAP	22	−0.1	0.3	
	NCV	dPNP	47	−0.4	0.2	n.s.
		TTR‐FAP	21	0.5	3.5	
Peroneal N. Mot.	Latency	dPNP	26	0.3	0.4	n.s.
		TTR‐FAP	15	−1.5	1.0	
	Amplitude	dPNP	26	−0.8	0.2	n.s.
		TTR‐FAP	15	−1.4	0.9	
	NCV	dPNP	26	−0.5	0.2	n.s.
		TTR‐FAP	12	1.7	2.8	
Tibial N. Mot.	Latency	dPNP	43	−0.7	0.3	n.s.
		TTR‐FAP	4	−0.7	0.4	
	Amplitude	dPNP	43	−0.3	0.3	n.s.
		TTR‐FAP	4	−1.3	0.7	
	NCV	dPNP	39	−0.5	0.2	n.s.
		TTR‐FAP	4	0.8	1.5	
Sural N. Sens.	Amplitude	dPNP	25	−2.0	0.1	n.s.
		TTR‐FAP	14	−1.7	0.2	
	NCV	dPNP	25	−3.3	0.4	*
		TTR‐FAP	13	−1.6	0.9	

#### 3.1.4 Autonomic function tests

**Figure 2:**
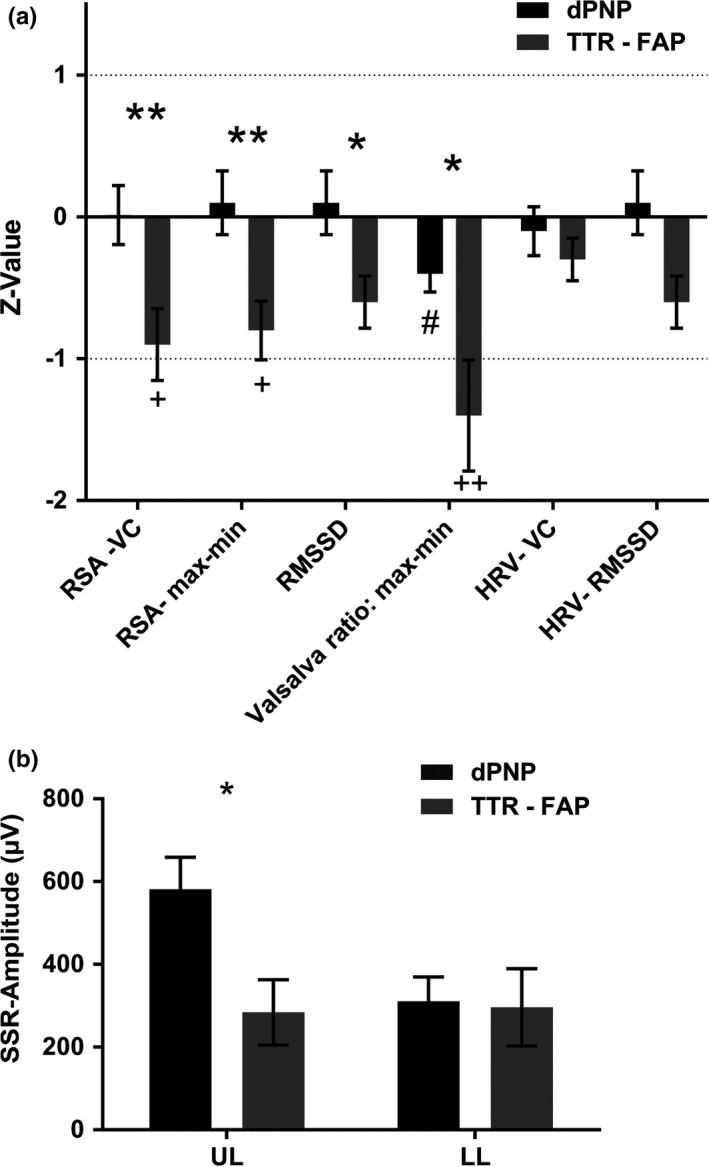
Results of the autonomic function test: (a) Summary of the results of the phenotypical comparison by autonomic function tests (AFT). Significant differences between TTR‐FAP and dPNP are indicated by * (*p < .05; **p < .01; ***p < .001); significant differences between dPNP and a normal population are indicated by # (^#^ *p* < .05; ^##^ *p* < .01; ^###^ *p* < .001); and significant differences between TTR‐FAP and a normal population are indicated by + (^+^ *p* < .05; ^++^ *p* < .01; ^+++^ *p* < .001). The Valsalva ratio was shown to be pathological in both groups when compared to a normal population. In the TTR‐FAP group, we also found pathological values for RSA‐vital capacity (RSA‐VC) and RSA‐max‐min. Significant differences between both groups were shown, meaning less response of the respiratory sinus arrhythmia in patients with TTR‐FAP. (b) The amplitude of the SSR in the upper limb (UL) was shown to be more diminished in TTR‐FAP, while it was comparable when measuring in the lower limb (LL)

Heart rate variability could be measured in 16 TTR‐FAP and 41 dPNP patients. Dropouts were due to cardiac arrhythmia which precludes a meaningful analysis. Pathological values were found in both groups, but TTR‐FAP patients were significantly more impaired compared to dPNP patients (see Figure 2a for details). At the arms but not at the feet, the SSR amplitude was lower in TTR‐FAP (284 μV TTR‐FAP; 581 μV dPNP; *p* = .022) (Figure 2b).

#### 3.1.5 Multivariate analysis

Prerequisites for variables to become included into the stepwise discriminant analysis (minimize Wilk's lambda) were (i) a firm scientific rationale, (ii) differences between TTR‐FAP and dPNP in the univariate tests (see above), or (iii) a reasonable number of valid data to prevent loss of too many cases. Included were as follows: CDT and WDT (hand) as small fiber tests, MPT (hand) as a surrogate of pain sensitization, the SSR at the upper limb as an autonomic test, and motor and sensory NCV of the ulnar nerve as a large fiber test. The HRV was excluded because of too many dropouts due to cardiac arrhythmias. As clinical parameters, we included the NIS‐LL, NIS‐UL, and the sum score of muscle weakness. Missing values were excluded listwise, 56 patients remained in the analysis.

The stepwise discriminant analysis selected the CDT, the MPT, and the sensory NCV of the ulnar nerve as the discriminant variables sufficient to discriminate TTR‐FAP from dPNP (Wilk's lambda = 0.61). The other variables did not contribute. Fisher's linear discriminant function was.

D = −1.1 + 0.35 *CDT − 0.30*MPT + 0.34*(ulnar SNCV).

This formula correctly classified the cases in 82.0% (see Table S2).

To test the validity of this analysis, TTR‐FAP patients were compared to CIN (*n* = 48 in analysis) or CIDP (*n* = 28 in analysis, only sensory NCS of the ulnar nerve, QST data were missing). CDT, MPT, and ulnar sensory NCV were able to classify 86.7% of the TTR‐FAP versus CIN patients correctly (Wilk′s lambda = 0.59; D = 0.90 + 0.327*CDT − 0.37*MPT + 0.28*(ulnar SNCV). Ulnar nerve sensory NCV alone correctly classified 67.9% of TTR‐FAP vs CIDP (D = 1.34 + 0.22*[ulnar SNCV]).

## 4 DISCUSSION

TTR‐FAP and dPNP both present with motor, sensory, and autonomic neuropathy. In our study, TTR‐FAP mainly differed from dPNP by abnormalities on the upper but not on the lower limbs. Discriminant analyses are widely used in recent bioinformatics analyses of cohort studies (McCarty, Punjabi, Kim, Frilot, & Marino, 2014). They allow the identification of predictors for group membership, especially if they are based on metric‐independent variables such as *z*‐scores as used in our study (Joseph, Hair, Barry, & Rolph, 2007). Fisher's nonstandardized linear discriminant function allows the exact group allocation of individual patients based on the independent variable in the analysis. The stepwise forward method “minimize Wilk's lambda” excludes variables with small discriminative power differences or high correlation. Thereby, CDT and MPT (hand) and ulnar SNCV were selected as the most important classifiers. These routine investigations alone allowed differentiation between TTR‐FAP and dPNP or CIN, and this discrimination was possible even though the TTR‐FAP group was genetically heterogeneous. Ulnar SNCV could also, although only to a lesser extend, discriminate between TTR‐FAP and CIDP. Thus, the combination of slow ulnar SNCV, insensitivity to cold, and hyperalgesia to toothpicks at the hands should alert physicians for amyloidosis.

Standard work‐up of polyneuropathy usually includes a complete medical history, a detailed clinical neurological examination, nerve conduction studies, and routine laboratory tests (Lubec, Mullbacher, Finsterer, & Mamoli, 1999). Thereby, the etiology can be identified in about 75% (Visser, Notermans, Linssen, van den Berg, & Vrancken, 2015). Usually, screening for inherited amyloidosis is not included. We now found that the aforementioned pattern of findings suggest TTR‐FAP. As expected, the discrimination against CIDP was worst because TTR‐FAP shares more clinical and neurophysiological similarities with CIDP than with dPNP or CIN (Mathis, Magy, Diallo, Boukhris, & Vallat, 2012; Plante‐Bordeneuve, 2014). These similarities are twofold: (i) Both, TTR‐FAP and CIDP, are not strictly length dependent and early affect the upper limbs (Plante‐Bordeneuve et al., 2007), (ii) TTR‐FAP and CIDP early cause motor symptoms (Plante‐Bordeneuve et al., 2007) while dPNP (and CIN) has a predominant sensory phenotype (Geber et al., 2013). This explains why the NDS, which reflects sensory and reflex findings, is higher in dPNP, whereas the NIS, which is driven by muscle weakness, is higher in TTR‐FAP, and might also explain that particularly the investigation of the upper limb segregates TTR‐FAP from dPNP (and CIN): QST proved differences in cold detection and pinprick perception, and the SSR was impaired. Investigations on the feet were less discriminative because their pathological findings can be expected in any moderately advanced neuropathy.

The early involvement of the upper limb in TTR‐FAP could have different explanations. First, TTR‐FAP progresses faster and thus affects the upper limb earlier after diagnosis than dPNP (Adams et al., 2015), even if dPNP is present for 5 years as in our study. Second, unlike dPNP, TTR‐FAP is not exclusively a dying‐back axonopathy but has also a neuronopathic component. Although pathological findings indicate a length‐dependent axonal neuropathy (Said et al., 1984) amyloid deposits with macrophage activation have been observed in the dorsal root ganglia and in the paravertebral sympathetic ganglia (Verghese, Bradley, Nemni, & McAdam, 1983). Furthermore, amyloid deposits in the peripheral nerves and blood vessels have rather a patchy than an even distribution (McKenzie et al., 2017). Both mechanisms might cause the early involvement of “short” nerve fibers in the upper limbs.

The neurophysiological findings in our TTR‐FAP and dPNP patients are representative and confirm previous investigations (Kim, Zeldenrust, Low, & Dyck, 2009). In particular, the impairment of warm and cold perception at the hands occurs early in TTR‐FAP (Plante‐Bordeneuve & Said, 2011). Furthermore, the hyperalgesia to pinprick stimuli on the hands is remarkable because the pattern of QST in common neuropathies like dPNP does only rarely include signs of hypersensitivity (Maier et al., 2010). Though speculative, the most likely explanation in TTR‐FAP is the sensitization of peripheral and central spinal cord nociceptive neurons by ongoing degeneration/regeneration involving inflammation, trophic factors, and glial activation in the dorsal horn (Sousa & Saraiva, 2003). The predominant electrophysiological pattern of both dPNP and TTR‐FAP in our study is axonal (Dyck, Lais, Karnes, O'Brien, & Rizza, 1986). However, demyelination has also been reported in TTR‐FAP (Plante‐Bordeneuve et al., 2007) and in CIDP (Cappelen‐Smith, Kuwabara, Lin, Mogyoros, & Burke, 2001). This might be another reason why TTR‐FAP can be misdiagnosed as CIDP. Autonomic dysfunction is an early finding in amyloid polyneuropathies (Plante‐Bordeneuve et al., 2007) and occurs earlier than in dPNP (Ewing, Campbell, & Clarke, 1980; Low et al., 2004), which is reflected by our findings.

One limitation of our study is the relatively small TTR‐FAP cohort because TTR‐FAP is rare in Germany. However, the cross‐validation of our results against CIN and CIDP significantly increases the credibility of the analysis. Furthermore, patients’ age varied between groups. However, *z*‐scores were calculated against age‐adjusted normal values. Therefore, age could only influence the comparison of clinical scores and the SSR, in which there are no normal values. However, neuropathic symptoms were more severe in TTR‐FAP patients although they were younger. This means that “age” as a source of variability seems to be of minor importance. Young age might even help to identify TTR‐FAP. A further source of variability might be that most (79%) but not all TTR‐FAP patients had the Val30Met mutation. Some mutations (e.g., Ile122Val) may show less polyneuropathic/demyelinating features. However, all patients included in this study were presented with polyneuropathy.

Finally, our results are valid in distal symmetrical polyneuropathies only. Our results cannot be transferred to neuropathies with asymmetrical clinical manifestation (i.e., “multiplex‐type”).

In conclusion, our results offer “a tool‐box”, that is, testing the upper limbs by NCV, QST, and SSR, to facilitate early identification of TTR‐FAP. In particular, cold and pinprick sensitivity at the hands, and the ulnar sensory NCV seem to be useful for the early detection of TTR‐FAP. Of note, these parameters are part of the standard work‐up of polyneuropathies (NCV) or can be easily implemented in the routine work‐up as “bed‐side‐tests” (insensitivity to cold assessed by cold reflex hammer or thermorollers; mechanical hyperalgesia to toothpicks at the hands) (Hansson, Backonja, & Bouhassira, 2007). We want to stress that a careful and comprehensive clinical neurological examination is essential in the work‐up of polyneuropathies, but that these three parameters should be paid special attention to, in order to discriminate TTR‐FAP from other polyneuropathies. The next step is to identify a better control group, that is, patients with neuropathy of unknown origin with the same biographic characteristics to refine the results of our study and foster the early diagnosis and treatment of TTR‐FAP. This will significantly improve prognosis by preventing irreversible nerve fiber damage.

## CONFLICT OF INTEREST

FEL reports no disclosures. APB received honoraria from serving on the scientific advisory board of Pfizer (last time 2015). CG reports no disclosures. FB reports funding by an unrestricted educational grant from Pfizer, Germany (WI201057, EFA).

## AUTHORS’ CONTRIBUTIONS

Fabiola Escolano‐Lozano, MD involved in study concept and design, wrote manuscript, performed data analysis and interpretation, patient recruitment and examination; Ana Paula Barreiros, MD wrote manuscript and involved in patient recruitment; Christian Geber, MD involved in study concept and design, wrote manuscript, patient recruitment, and examination; Frank Birklein, MD involved in study concept and design, wrote manuscript, performed data analysis and interpretation, patient recruitment, and examination.
