# Supplementary material for: Transthyretin familial amyloid polyneuropathy (TTR‐FAP): Parameters for early diagnosis
Source: Brain Behav. 2017 Dec 19;8(1):e00889. doi: 10.1002/brb3.889 (PMC5853640; doi:10.1002/brb3.889)
Supplement: Supplementary file 1 [file BRB3-8-e00889-s001.docx]

**Transthyretin familial** **Amyloid Polyneuropathy (TTR-FAP): Parameters for early diagnosis** (87characters)

Fabiola Escolano-Lozano, MD ^1^, Ana Paula Barreiros, MD ^2^, Frank Birklein, MD ^1^*^+,^, Christian Geber, MD ^1,3+^

^1^ Department of Neurology, University Medical Center of the Johannes Gutenberg University Mainz, Mainz Germany

^2^ German Association for Liver Transplantation, Mainz, Germany

^3^ Red Cross Pain Center, Mainz, Germany

*corresponding author, ^+^shared senior authorship

**Supplemental data**

| QST comprises 13 thermal and mechanical detection and pain thresholds | | | | | |  |  |
| --- | --- | --- | --- | --- | --- | --- | --- |
|  | Thermal detection thresholds for the perception of cold (CDT),   warm (WDT) and paradoxical heat sensations (PHS)   during alternating warm and cold stimuli (thermal sensory limen, TSL) | | | | | |  |
|  |  |  |  |  |  |  |  |
|  |  |  |  |  |  |  |  |
|  |  |  |  |  |  |  |  |
|  | Thermal pain thresholds for cold (CPT) and hot stimuli (HPT),   mechanical detection thresholds for touch (MDT) and vibration (VDT) | | | | | |  |
|  |  |  |  |  |  |  |  |
|  |  |  |  |  |  |  |  |
|  | Mechanical pain sensitivity including thresholds for pinprick (MPT)   and blunt pressure (PPT), stimulus/response-functions for pinprick   sensitivity (MPS) and dynamic mechanical allodynia (DMA), and pain summation  to repetitive pinprick stimuli (wind-up like pain, WUR). | | | | | | |
|  |  |  |  |  |  |  |  |
|  |  |  |  |  |  |  |  |
|  |  |  |  |  |  |  |  |
|  |  |  |  |  |  |  |  |
|  |  | | | | | | |
|  |  |  |  |  |  |  |  |

Supp. 1: Description of QST parameter

|  |  | **Predicted group** | |
| --- | --- | --- | --- |
|  | **Diagnosis** | dPNP | TTR-FAP |
| **Number** | dPNP | 33 | 5 |
|  | TTR-FAP | 4 | 8 |
| **%** | dPNP | 86.8 | 13.2 |
|  | TTR-FAP | 33.3 | 66.7 |

Suppl. Table 2: Results of the discriminant analysis. Considering the SNCV of the ulnar nerve, correct classification of the patients was possible in 82% of the cases.
